# Non-Hodgkin lymphoma response evaluation with MRI texture classification

**DOI:** 10.1186/1756-9966-28-87

**Published:** 2009-06-22

**Authors:** Lara CV Harrison, Tiina Luukkaala, Hannu Pertovaara, Tuomas O Saarinen, Tomi T Heinonen, Ritva Järvenpää, Seppo Soimakallio, Pirkko-Liisa I Kellokumpu-Lehtinen, Hannu J Eskola, Prasun Dastidar

**Affiliations:** 1 Tampere University Medical School, Tampere, Finland; 2 Department of Biomedical Engineering, Tampere University of Technology, Tampere, Finland; 3 Science Center, Pirkanmaa Hospital District, Tampere, Finland; 4 Tampere School of Public Health, University of Tampere, Tampere, Finland; 5 Department of Oncology, Tampere University Hospital, Tampere, Finland; 6 Department of Radiology, Regional Imaging Centre, Pirkanmaa Hospital District, Tampere, Finland

## Abstract

**Background:**

To show magnetic resonance imaging (MRI) texture appearance change in non-Hodgkin lymphoma (NHL) during treatment with response controlled by quantitative volume analysis.

**Methods:**

A total of 19 patients having NHL with an evaluable lymphoma lesion were scanned at three imaging timepoints with 1.5T device during clinical treatment evaluation. Texture characteristics of images were analyzed and classified with MaZda application and statistical tests.

**Results:**

NHL tissue MRI texture imaged before treatment and under chemotherapy was classified within several subgroups, showing best discrimination with 96% correct classification in non-linear discriminant analysis of T2-weighted images.

Texture parameters of MRI data were successfully tested with statistical tests to assess the impact of the separability of the parameters in evaluating chemotherapy response in lymphoma tissue.

**Conclusion:**

Texture characteristics of MRI data were classified successfully; this proved texture analysis to be potential quantitative means of representing lymphoma tissue changes during chemotherapy response monitoring.

## Background

Quantitative image analysis may provide new clinically relevant information on the target of interest, constituting a major advantage in clinical work as well as in research. The most significant objectives in quantitative image analysis are to find tissue-characterizing features with biological significance and which correlate with pathophysiology detected by other methods, i.e. clinical examination, other imaging modalities and pathological-anatomical diagnosis, and secondly to provide this new information on the properties of tissues to be used alone or in combination with other clinical information allowing more reliable detection of disease and sophisticated tissue classification as a clinical diagnostic and follow-up tool.

Precise and earlier diagnostics and monitoring treatment response are significant both for the individual patient's prognosis and on a larger scale in developing treatment procedures, especially in malignant diseases. Within the research on solid tumors extensive and widely used Response Evaluation Criteria in Solid Tumors (RECIST) Guidelines may be followed to obtain intra- and inter center comparable results. RECIST defines measurability of tumor lesions and specifies methods of measurements with different techniques [1]. According to the RECIST criteria measure of tumor response from radiological images is done by measuring lesions one-dimensionally, furthermore the World Health Organization (WHO) criteria use two dimensional analysis and several research groups volumetric three-dimensional analysis [2].

Staging of non-Hodgkin's lymphomas (NHL) is the key element of treatment planning for this heterogeneous group of malignancies. A variety of diagnostic tools, including biopsies, computed tomography (CT), magnetic resonance imaging (MRI), ^18^F-fluorodeoxyglucose positron emission tomography (FDG-PET) or molecular markers are used in pre-treatment staging [3]. Enhancement with contrast media could also help the evaluation in using different imaging modalities. The same tools are applied to evaluate the response to different types of treatment. Novel techniques such as hybrid positron emission tomography – computed tomography (PET-CT) imaging and new PET tracers like ^18^F-fluoro-thymidine (^18^F-FLT) may increase the sensitivity of response assessment [4]. Reports aiming international standardization of clinical response criteria for NHL have been published [5,6], and these criteria are in wide clinical use. A combination of cyclophosphamide, doxorubicin, vincristine and prednisone (CHOP) remains the mainstay of therapy. The addition of a chimeric-anti-CD20 immunoglobulin G1 monoclonal antibody, rituximab (Mabthera^®^), has resulted in a dramatic improvement in the outcome of the most common NHL, diffuse large B-cell lymphoma, but has also been shown to effective in other type of B-cell lymphomas [7-9].

Several quantitative MRI studies have indicated that texture analysis (TA) has the ability to detect differences between tissues and subtle changes between disease burden and normal tissue. Successful applications of TA have been reported from studying neurological diseases [10-15], brain tumors [16,17], amygdale activation [18], muscles [19,20], trabecular bone [21-23], liver [24-26], breast cancer [27-31] and lymphomas [32].

In this paper we report the ability of TA to detect changes in NHL solid tissue masses during chemotherapy. The change in texture appearance is controlled by quantitative volumetric analysis. We classify statistical, autoregressive (AR-) model and wavelet texture parameters representing pre-treatment and two under chemotherapy stages of tumors with four analyses: raw data analysis (RDA), principal component analysis (PCA), linear (LDA) and non-linear discriminant analysis (NDA). The final objective is to show that these texture parameters of MRI data can be successfully tested with Wilcoxon paired test and Repeatability and Reproducibility (R&R) test for assess the impact of the parameters usability in evaluating chemotherapy response in lymphoma tissue.

## Methods

Tumor Response Evaluation (TRE) is a wide prospective clinical project ongoing at our university hospital on cancer patients, where tumor response to treatment is evaluated and followed up using simultaneously CT, MRI and PET imaging methods. Clinical responses for these lymphoma patients were assessed according to the guidelines of the international working group response criteria. In this texture analysis study, as a part of extensive project, the focus was on quantitative imaging methods and only the response in predefined solid NHL masses was evaluated. The ethics committee of the hospital approved the study and participants provided written informed consent. Primary inclusion criteria were NHL patients with at least one bulky lesion (over 3 centimeters) coming for curative aimed treatment. Exclusion criteria were central nervous disease, congestive heart failure New York Heart Association Classification (NYHA) III-IV, serious psychiatric disease, HIV infection and pregnancy.

### Patients

MRI images of nineteen NHL patients participating in the TRE project were selected for the first part of this study. One of these patients was excluded due to the smaller amount of image data from the second part analyses. There were 14 male and 5 female patients aged 34–75. These patients had untreated or relapsed histologically diagnosed high/intermediate (N = 8, 42%) or low-grade (N = 11, 58%) NHL with an evaluable lymphoma lesion either in the abdominal area (N = 16) or in the clavicular and axillary lymph node area (N = 3). The treatment given was chemotherapy alone or combined with humanized antibody, rituximab (Mabthera^®^). Therapy regimens were CHOP (N = 5), R-CHOP (rituximab and CHOP) (N = 8), and CVP (cyclophosphamide, vincristine and prednisone) (N = 1), CHOP-like CNOP (cyclophosphamide, mitoxantrone, vincristine and prednisone) (N = 1), ChlP (chlorambucil and prednisone) (N = 1), starting with CHOP and changing to R-CHOP (N = 2), starting with R-CHOP and changing to R-CVP (N = 1). Chemotherapy regimens were selected according to patients' clinical status. Chemotherapy courses were repeated every three weeks, and 4 to 9 courses were given according to clinical response. Two patients received 4 cycles, four patients 6 cycles, one patient 7 cycles, and 11 received 8 cycles, and one 9 cycles.

### MR imaging schedule

**Figure 1:**
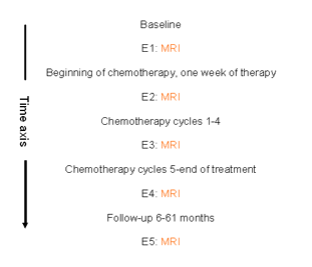
**Time frame of the study**. E1-E5 refers to the MRI examination timepoints 1–5, respectively.

MR imaging in clinical practice as well as in this study was carried out at staging phase before any treatment (examination 1, E1), after the first chemotherapy cycle (examination 2, E2), and after the fourth chemotherapy cycle (examination 3, E3). In addition patients were followed up by using MRI six months and 6–61 months after the completion of therapy. The time frame of the study is presented in Figure 1.

### MR image acquisition

Imaging was performed on a 1.5 T MRI device (GE Signa, Wisconsin, USA).

One contrast enhanced sequence acquired from the first and second imaging timepoint were included for volume analysis of lymphoma masses. The sequence used was axial T2-weighted fast spin echo (FSE) fat saturation (FAT SAT) sequence (TR 620 ms, TE 10 ms), with intravenous contrast agent gadolinium chelate (gadobenate dimeglumine, 0.2 mg/ml, 10 ml), slice thickness ranged from 5 mm to 12 mm.

One or two T1- and T2-weighted axial image serquences from the first three imaging timepoints of every patient were taken for texture analysis. The T1-weighted series comprised T1-weighted spin echo (SE) and T1-weighted SE FAT SAT sequences (TR 320–700 ms, TE 10 ms), the T2-weighted sequences were FSE FAT SAT (TR 3 320–10 909 ms, TE 96 ms). Repetition time TR varied between and within patients. Slice thickness varied between patients according to clinical status from 5 mm to 12 mm; most patients had two different slice thickness series, the general combination was 5 mm and 8 mm series. Pixel size varied from 1.33 mm*1.33 mm to 1.80 mm*1.80 mm, and a 256*256 matrix was used.

### Texture analysis with MaZda

**Figure 2:**
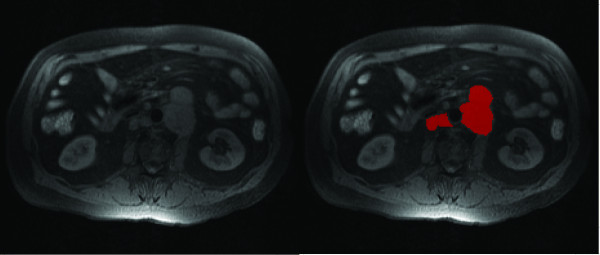
**Axial T1-weighted fat saturation image slice of the abdomen of a typical subject (left), and ROI drawn on lymphoma mass (right)**.

Texture parameter calculation was the first stage of the texture analyses. Stand-alone DICOM viewer application was used to select three to five slices from every image series for analysis. Region of interest (ROI) setting and texture analysis were carried out with MaZda software (MaZda 3.20, The Technical University of Lodz, Institute of Electronics) [33,34]. The lymphoma masses were manually selected and set as ROIs (Figure 2). Texture features calculated were based on histogram, gradient, run-length matrix, co-occurrence matrix, autoregressive model and wavelet-derived parameters [34]. Image grey level intensity normalization computation separately for each ROI was performed with method limiting image intensities in the range [μ-3σ, μ+3σ], where μ is the mean grey level value and σ the standard deviation. This method has been shown to enhance differences between two classes when comparing image intensity normalization methods in texture classification [35].

Fisher coefficient (Fisher) and classification error probability (POE) combined with average correlation coefficients (ACC) provided by MaZda were used to identify the most significant texture features to discriminate and classify the three evaluation stages of lymphoma tissue. Ten texture features were chosen by both methods (Fisher, POE+ACC). This feature selection was performed separately for the T1- and T2-weighted image sets. In these subgroups feature selection was run for the following imaging stages: combination of all imaging timepoints (E1, E2, and E3), and all combinations of the two aforementioned. Slice thickness was not taken into account.

### Volumetric analysis

The volumetry of the solid lymphoma masses was evaluated between diagnostic stage (E1) and after the first treatment (E2). The masses were selected for evaluation before chemotherapy. The same masses were followed after the first treatment. Volumetric analysis based on MRI images was performed with semiautomatic segmentation software Anatomatic™ [36] with region growing method. [37].

### Clinical parameters analyses

The patients' subjective views on their clinical symptoms was observed between two stages: at the diagnosis and after the first treatment. The subjective views were set in two groups: symptoms unchanged or relieved.

Grade of malignity was classed into two groups: 1) low; 2) high/intermediate.

### Tissue classification

B11 application (version 3.4) of MaZda software package was used for texture data analysis and classification. Analyses were run between all combinations of imaging stages separately for T1- and T2-weighted images. Analyses were performed for combination of parameters selected automatically with Fisher and POE+ACC methods for 1) the specific imaging timepoint pair in question and 2) for all imaging stages in particular image type (T1-, T2-weighted). Feature standardization was used in B11, the mean value being subtracted from each feature and the result divided by the standard deviation. Raw data analysis (RDA), principal component analysis (PCA), and linear (LDA) and nonlinear discriminant analysis (NDA) were run for each subset of images and chosen texture feature groups. B11 default neural network parameters were used. Nearest-neighbor (1-NN) classification was performed for the raw data, the most expressive features resulting from PCA and the most discriminating features resulting from LDA. Nonlinear discriminant analysis carried out the classification of the features by artificial neural network (ANN). These classification procedures were run by B11 automatically.

### Statistical analyses

Statistical analyses were run for the texture features MaZda's automatic methods (Fisher and POE+ACC) had shown to give best discrimination between imaging timepoints. The T1- and T2-weighted image texture parameters were tested separately. Texture parameters for 18 patients were included in the test, one patient participating in MaZda texture parameter calculation was excluded because of smaller amount of image data than other patients leading to reduced textural data.

In analyzing and seeking the best parameters for classification, it is vital to ensure low overall variation in the treatment process and to ascertain how this variation can be focused onto different components in the whole process. In the present study the repeatability and reproducibility (R&R) method was applied. The design of the study was experimental, the aim being to estimate different sources of variation in the lymphoma texture at the three different timepoints (examinations 1, 2, and 3) and repeating the same measurements three times. Because the distributions were skewed, the range method was used.

According to the standard Gage R&R terminology timepoints stand for operators, patients for parts and repeated measurements for trials. In statistical terms the following variance components were estimated: repeatability (difference across measurements), reproducibility (difference across timepoints) and variability (difference across patients). Repeatability describes intrapatient variation, i.e., how a given measurer repeats the same planning process. Reproducibility describes interpatient variation, i.e., how different measurements at the timepoints follow the same planning process and variability describes interpatient variation, i.e. how well the same physician can repeat the planning process for different kinds of patients. The total error – also known as the combined R&R effect – includes repeatability and reproducibility, and only patient-to-patient variation is excluded. In industrial applications the combined R&R should not exceed 10% of the total variation, but in certain situations a total error up to 30% may be acceptable. The present statistical analyses were performed by Statistica/W (Version 5.1, 98 edition, Statsoft. Inc, Tulsa, OK, USA).

Textural data from T1- and T2-weighted fat saturation image series were analysed separately and both groups divided into two subgroups according to slice thickness: 5–7 mm and 8–12 mm. Differences between imaging timepoints were analysed by Wilcoxon Signed Ranks.

Mann-Whitney test was used to test rank parameters grouped by grade of malignity and subjective change of symptoms. These analyses were performed by SPSS for Windows, version 14.0.2.

## Results

### Volumetric analysis

The median volume of the lymphoma masses before treatment (E1) was 429 cm^3^, ranging from 72 cm^3 ^to 2144 cm^3^. The median volume of the masses calculated from the second imaging timepoint (E2) was 190 cm3, ranging from 30 cm^3 ^to1622 cm^3^. After the first treatment cycle, the lymphoma mass volume had decreased in all patients. The median decline in volume was 32%, ranging from 3% to 76%. The results of this volumetric analysis have been published earlier in more detail [37]. The volumetry results of the first and second imaging are given in cm^3^, and the volume change is calculated in percentages in Table 1.

**Table 1:** Grade of malignancy (1 = low, 2 = high/intermediate), subjective view of change in symptoms between pretreatment stage (E1) and after first chemotherapy cycle (E2) (0 = unchanged, 1 = relieved).

**Patient**	**Grade of malignity**	**Symptoms**	**Volume**		
	1 = low 2 = high/intermediate	0 = unchanged 1 = relieved	E1 (cm3)	E2 (cm3)	Change%
1	2	1	429	105	-76%
2	2	1	183	64	-65%
3	1	1	173	66	-62%
4	1	1	529	459	-13%
5	1	0	570	419	-26%
6	1	1	800	595	-26%
7	2	1	146	118	-19%
8	2	0	118	80	-32%
9	1	1	367	246	-33%
10	1	0	850	769	-10%
11	2	1	2144	1622	-24%
12	2	1	72	30	-58%
13	2	0	140	52	-63%
14	2	1	274	93	-66%
15	1	1	795	190	-76%
16	1	0	824	797	-3%
17	1	0	750	579	-23%
18	1	0	273	66	-76%
19	1	0	771	522	-32%

Results of the volumetric analysis of first (E1) and second imaging stages (E2). Volumes are given in cm^3^, and the volume change calculated in percentages.

### Clinical parameters analyses

According to the patient's subjective estimates clinical symptoms between first and second imaging timepoint were unchanged in eight patients and relieved in 11 patients. Grades of malignancy and subjective view on symptoms are presented in Table 1 with volumetry results.

### Texture data: MaZda and B11 analyses

We included in the analyses 108 T1-weighted and 113 T2-weighted images from E1; 103 T1-weighted and 105 T2-weighted images from E2; and 97 T1-weighted images and 99 T2-weighted images from E3.

Texture features were selected with Fisher and POE+ACC methods in MaZda from 300 original parameters calculated for each of the four subgroups in both image data classes T1- and T2-weighted.

We found that the most significant features varied clearly between imaging stages. The whole of 74 TA features ranked first to tenth significant feature in tested subgroups. There were three histogram parameters, 55 co-occurrence parameters, nine run-length parameters, four absolute gradient parameters and three autoregressive model parameters. No wavelet parameters were placed in the top group.

Data analyses RDA, PCA, LDA and NDA show texture changes between imaging points. The analyses did not perform well the task of discriminating all three imaging timepoints (E1, E2, E3) at same time. Slightly better classification was achieved between the first and second examinations, and between the second and third examinations. The method was successful in classifying the textural data achieved from the pre-treatment and third imaging timepoints, the best discrimination was obtained within T2-weighted leading to NDA classification error of 4%, and within T1-weighted NDA 5% error. Classification of different examination stages lead to same level results in T1- and T2-weighted images. The overall classification results are presented in Table 2 and Table 3.

**Table 2:** MaZda classification results – results obtained within T1-weighted images.

**T1-weighted images classification**		**RDA**	**PCA**	**LDA**	**NDA**
**Examinations**		mis%	mis%	mis%	mis%
E1, E2, E3	Combination E1, E2, E3	36%	34%	46%	31%
					
E1, E2	Combination E1, E2, E3	36%	34%	46%	31%
	Combination E1, E2	24%	26%	34%	16%
					
E1, E3	Combination E1, E2, E3	18%	18%	13%	6%
	Combination E1, E3	17%	17%	15%	5%
					
E2, E3	Combination E1, E2, E3	26%	26%	34%	18%
	Combination E2, E3	25%	27%	30%	13%

Imaging timepoint (E1, E2, E3) combinations for classification analyses. Feature selection methods given in rows. Misclassification percentage (mis%) given for raw data analysis (RDA), principal component analysis (PCA), linear discriminant analysis (LDA) and non-linear discriminant analysis (NDA) in columns. "Combination E1, E2, E3" in feature selection methods refers to features, which have proved to give best discrimination in all imaging timepoints analyses with Fisher and POE+ACC methods, combination of two imaging timepoints refers respectively to features from the analyses in question.

**Table 3:** MaZda classification results – results in groups of T2-weighted images.

**T2-weighted images classification**		**RDA**	**PCA**	**LDA**	**NDA**
**Examinations**	**Feature selection method**	mis%	mis%	mis%	mis%
E1, E2, E3	Combination E1, E2, E3	34%	35%	47%	30%
					
E1, E2	Combination E1, E2, E3	29%	29%	39%	19%
	Combination E1, E2	37%	35%	40%	35%
					
E1, E3	Combination E1, E2, E3	15%	14%	19%	4%
	Combination E1, E3	16%	17%	21%	4%
					
E2, E3	Combination E1, E2, E3	25%	24%	25%	14%
	Combination E2, E3	24%	23%	30%	12%

Imaging timepoint (E1, E2, E3) combinations for classification analyses. Feature selection methods given in rows. Misclassification percentage (mis%) given for raw data analysis (RDA), principal component analysis (PCA), linear discriminant analysis (LDA) and non-linear discriminant analysis (NDA) in columns. "Combination E1, E2, E3" in feature selection methods refers to features, which have proved to give best discrimination in all imaging timepoints analyses with Fisher and POE+ACC methods, combination of two imaging timepoints refers respectively to features from the analyses in question.

### Texture data: Statistical analyses

The values of 73 features obtained with MaZda feature selection methods were tested with Wilcoxon paired test for groups obtained from imaging timepoints a) E1 and E2, b) E2 and E3, c) E1 and E3. T1- and T2-weighted fat saturation image series data were set as their own groups and further into two subgroups according to slice thickness: 5–7 mm and 8–12 mm.

R&R test parameter repeatability was used to describe the variation in texture features between image slices within imaging sequence, and parameter reproducibility to describe the variation between examination stages. This test was performed separately for T1- and T2-weighted images in all three combinations of two imaging points. Differences in slice thickness were not taken into account. Reproducibility values were expected to be quite large because the aim was that the treatment given between imaging stages would take effect and be shown in image texture. In contrast, repeatability values (i.e. differences between images taken at the same timepoint) were expected to be zero. There is no exact expected ratio for reproducibility and patient-to-patient variation in such studies and thus no exact value for percentage of reproducibility, so that the difference between different imaging stages was significant.

The texture parameters giving the best discrimination within T1-weighted image groups in two imaging stage comparison are given in Table 4, Table 5 and Table 6; and respectively for T2-weighted image groups in Table 7, Table 8 and Table 9. Reproducibility percentage and Repeatability percentage of the total are given for all parameters. Wilcoxon paired test p-values are given for all parameters for separate groups regarding slice thickness (groups 5–7 mm and 8–12 mm).

**Table 4:** Summary table of texture parameters ranked 1-10 with Fisher and POE+ACC methods according to test subgroup T1-weighted images and imaging timepoints E1 and E2.

**T1-WEIGHTED IMAGES**	**R&R**	**R&R**	**Wilcoxon**	**Wilcoxon**
**E1-E2 analyses**	Repeatability % of total	Reproducibility % of total	Slice thickness <8 mm *p*	Slice thickness >= 8 mm *p*
**HISTOGRAM PARAMETERS**				
Percentile, 1%	15.349	0.069	0.286	0.672
**CO-OCCURENCE MATRIX PARAMETERS**				
Difference entropy S(1,0)	6.874	25.411	0.074	0.018
Difference entropy S(0,1)	7.725	26.783	0.074	0.028
Difference entropy S(1,1)	6.970	24.413	0.139	0.018
Difference entropy S(2,0)	8.409	28.186	0.114	0.018
Sum average S(0,2)	52.143	4.597	0.285	0.499
Difference entropy S(2,2)	11.265	22.824	0.093	0.018
Difference entropy S(3,0)	15.434	11.836	0.241	0.018
Angular second moment S(5,-5)	18.976	7.234	0.093	0.612
Sum of squares S(5,-5)	58.267	1.780	0.721	0.310
Sum average S(5,-5)	15.420	16.235	0.445	1.000
**RUN-LENGTH MATRIX PARAMETERS**				
Grey level nonuniformity, 0°	6.015	43.441	0.051	0.128
Grey level nonuniformity, 90°	8.822	35.055	0.028	0.091
Grey level nonuniformity, 45°	4.635	13.324	0.028	0.176
Grey level nonuniformity, 135°	4.734	39.630	0.037	0.249
**ABSOLUTE GRADIENT PARAMETERS**				
Variance	28.133	22.699	0.445	0.018
**AUTOREGRESSIVE MODEL PARAMETERS**				
Teta 2	65.193	2.741	0.575	0.237
Teta 4	66.319	2.285	0.575	0.398

Texture parameters are given in rows. In the columns R&R repeatability and reproducibility of total, and Wilcoxon test for fat saturation series grouped with image slice thickness less than 8 mm, and 8 mm or thicker.

**Table 5:** Summary table of texture parameters ranked 1-10 with Fisher and POE+ACC methods according to test subgroup T1-weighted images and imaging timepoints E2 and E3.

**T1-WEIGHTED IMAGES**	**R&R**	**R&R**	**Wilcoxon**	**Wilcoxon**
**E2-E3 analyses**	Repeatability % of total	Reproducibility % of total	Slice thickness <8 mm *p*	Slice thickness >= 8 mm *p*
**HISTOGRAM PARAMETERS**				
Variance	11.452	22.145	0.953	0.465
**CO-OCCURENCE MATRIX PARAMETERS**				
Contrast S(2,0)	31.815	28.807	0.139	0.465
Contrast S(3,0)	27.957	40.317	0.051	0.144
Difference variance S(3,0)	26.169	35.250	0.139	0.273
Contrast S(4,0)	29.032	37.330	0.051	0.144
Correlat S(4,0)	25.661	36.025	0.086	0.144
Correlat S(0,4)	21.528	38.249	0.139	0.068
Correlat S(5,0)	23.130	39.697	0.038	0.068
Sum average S(5,0)	55.837	4.961	0.214	0.144
Sum average S(0,5)	44.169	6.142	0.859	0.715
Inverse difference moment S(5,5)	53.397	24.684	0.678	0.465
Difference variance S(5,-5)	50.986	14.473	0.515	0.715
**RUN-LENGTH MATRIX PARAMETERS**				
Grey level nonuniformity, 0°	6.015	43.441	0.066	0.273
Run length nonuniformity, 45°	7.013	31.416	0.139	0.068
Grey level nonuniformity, 45°	4.635	13.324	0.066	0.465
Short run emphasis, 135°	13.062	21.630	0.021	0.144
**ABSOLUTE GRADIENT PARAMETERS**				
Mean	24.582	28.201	0.038	0.144
Kurtosis	60.387	1.194	0.767	1.000
**AUTOREGRESSIVE MODEL PARAMETERS**				
Teta 3	58.511	0.000	0.028	0.465

Texture parameters are given in rows. In the columns R&R repeatability and reproducibility of total, and Wilcoxon test for fat saturation series grouped with image slice thickness less than 8 mm, and 8 mm or thicker.

**Table 6:** Summary table of texture parameters ranked 1-10 with Fisher and POE+ACC methods according to test subgroup T1-weighted images and imaging timepoints E1 and E3.

**T1-WEIGHTED IMAGES**	**R&R**	**R&R**	**Wilcoxon**	**Wilcoxon**
**E1-E3 analyses**	Repeatability % of total	Reproducibility % of total	Slice thickness <8 mm *p*	Slice thickness >= 8 mm *p*
**HISTOGRAM PARAMETERS**				
MinNorm	24.793	2.445	0.504	0.465
Percentile, 1%	15.349	0.069	0.964	0.715
**CO-OCCURENCE MATRIX PARAMETERS**				
Inverse difference moment S(2,0)	20.950	29.298	0.008	0.068
Contrast S(3,0)	27.957	40.317	0.008	0.068
Correlation S(3,0)	24.569	38.395	0.021	0.068
Difference variance S(3,0)	26.169	35.250	0.021	0.068
Contrast S(4,0)	29.032	37.330	0.010	0.068
Correlation S(4,0)	25.661	36.025	0.021	0.068
Inverse difference moment S(4,0)	19.088	34.553	0.004	0.068
Correlation S(4,4)	17.730	40.414	0.021	0.068
Sum of squares S(4,-4)	52.253	2.218	0.859	1.000
Correlation S(5,0)	23.130	39.697	0.016	0.068
Inverse difference moment S(5,0)	23.111	37.188	0.013	0.068
Sum of squares S(0,5)	66.827	1.190	0.041	0.715
Sum of squares S(5,5)	64.191	3.647	0.477	0.715
**RUN-LENGTH MATRIX PARAMETERS**				
Grey level nonuniformity, 45°	4.635	13.324	0.003	0.068
Grey level nonuniformity, 135°	4.734	39.630	0.003	0.068
Fraction of image in runs, 135°	13.014	23.544	0.003	0.068

Texture parameters are given in rows. In the columns R&R repeatability and reproducibility of total, and Wilcoxon test for fat saturation series grouped with image slice thickness less than 8 mm, and 8 mm or thicker.

**Table 7:** Summary table of texture parameters ranked 1-10 with Fisher and POE+ACC methods according to test subgroup T2-weighted images and imaging timepoints E1 and E2.

**T2-WEIGHTED IMAGES**	**R&R**	**R&R**	**Wilcoxon**	**Wilcoxon**
**E1-E2 analyses**	Repeatability % of total	Reproducibility % of total	Slice thickness <8 mm *p*	Slice thickness >= 8 mm *p*
**HISTOGRAM PARAMETERS**				
MinNorm	14.090	24.380	0.861	0.636
**CO-OCCURENCE MATRIX PARAMETERS**				
Difference variance S(1,-1)	24.802	17.121	0.249	0.266
Sum average S(2,2)	38.483	23.527	0.552	0.163
Contrast S(3,0)	22.618	45.195	0.087	0.025
Contrast S(3,3)	23.282	48.345	0.152	0.102
Contrast S(4,0)	26.599	44.458	0.221	0.013
Contrast S(4,4)	31.083	41.015	0.116	0.049
Difference variance S(4,4)	35.305	32.674	0.196	0.019
Contrast S(4,-4)	40.897	22.850	0.013	0.266
Sum average S(4,-4)	10.802	1.906	0.345	0.210
Contrast S(5,0)	30.110	41.229	0.422	0.007
Sum of squares S(5,0)	64.138	7.335	0.807	0.076
Difference variance S(5,0)	34.811	32.369	0.917	0.009
Contrast S(0,5)	41.519	29.671	0.055	0.210
Contrast S(5,5)	39.461	38.040	0.133	0.102
Sum of squares S(5,5)	80.906	0.000	0.972	0.906
**RUN-LENGTH MATRIX PARAMETERS**				
Short run emphasis, 90°	10.659	12.516	0.087	0.149
Fraction of image in runs, 90°	11.662	12.685	0.101	0.124
**ABSOLUTE GRADIENT PARAMETERS**				
Mean	18.036	44.271	0.046	0.287
Skewness	63.599	15.598	0.382	0.492

Texture parameters are given in rows. In the columns R&R repeatability and reproducibility of total, and Wilcoxon test for fat saturation series grouped with image slice thickness less than 8 mm, and 8 mm or thicker.

**Table 8:** Summary table of texture parameters ranked 1-10 with Fisher and POE+ACC methods according to test subgroup T2-weighted images and imaging timepoints E2 and E3.

**T2-WEIGHTED IMAGES**	**R&R**	**R&R**	**Wilcoxon**	**Wilcoxon**
**E2-E3 analyses**	Repeatability % of total	Reproducibility % of total	Slice thickness <8 mm *p*	Slice thickness >= 8 mm *p*
**HISTOGRAM PARAMETERS**				
MinNorm	14.090	24.380	0.002	0.124
Variance	1.655	16.743	0.028	0.149
**CO-OCCURENCE MATRIX PARAMETERS**				
Contrast S(2,0)	19.563	41.264	0.055	0.001
Contrast S(2,2)	23.139	43.325	0.033	<0,001
Contrast S(3,0)	22.618	45.195	0.023	0.002
Correlation S(3,0)	21.555	40.965	0.009	0.001
Contrast S(0,3)	30.424	34.725	0.116	<0,001
Contrast S(3,3)	23.282	48.345	0.023	0.004
Correlation S(3,3)	22.095	44.779	0.016	0.010
Contrast S(4,0)	26.599	44.458	0.006	0.011
Correlation S(4,0)	23.479	41.166	0.003	0.009
Sum of squares S(4,0)	71.978	3.535	0.807	0.868
Correlation S(4,4)	23.823	42.301	0.016	0.055
Difference entropy S(4,-4)	10.347	7.011	0.039	0.210
Sum average S(0,5)	35.828	0.000	0.972	0.011
Angular second moment S(5,-5)	8.994	12.106	0.064	0.015
Inverse difference moment S(5,-5)	46.459	0.000	0.917	0.795
**RUN-LENGTH MATRIX PARAMETERS**				
Grey level nonuniformity, 135°	6.265	33.780	0.003	0.004
**ABSOLUTE GRADIENT PARAMETERS**				
Mean	18.036	44.271	0.039	<0,001
Skewness	63.599	15.598	0.221	0.044

Texture parameters are given in rows. In the columns R&R repeatability and reproducibility of total, and Wilcoxon test for fat saturation series grouped with image slice thickness less than 8 mm, and 8 mm or thicker.

**Table 9:** Summary table of texture parameters ranked 1-10 with Fisher and POE+ACC methods according to test subgroup T2-weighted images and imaging timepoints E1 and E3.

**T2-WEIGHTED IMAGES**	**R&R**	**R&R**	**Wilcoxon**	**Wilcoxon**
**E1-E3 analyses**	Repeatability % of total	Reproducibility % of total	Slice thickness <8 mm *p*	Slice thickness >= 8 mm *p*
**HISTOGRAM PARAMETERS**				
MinNorm	14.090	24.380	0.003	0.130
**CO-OCCURENCE MATRIX PARAMETERS**				
Contrast S(2,0)	19.563	41.264	0.011	0.001
Contrast S(2,2)	23.139	43.325	0.006	<0,001
Contrast S(3,0)	22.618	45.195	0.009	0.001
Correlation S(3,0)	21.555	40.965	0.007	0.001
Sum average S(3,0)	28.935	19.345	0.033	0.035
Contrast S(3,3)	23.282	48.345	0.006	<0,001
Correlation S(3,3)	22.095	44.779	0.007	<0,001
Sum average S(3,-3)	20.384	0.353	0.087	0.017
Contrast S(4,0)	26.599	44.458	0.007	0.001
Contrast S(4,4)	31.083	41.015	0.009	<0,001
Correlation S(4,4)	23.823	42.301	0.007	<0,001
Sum of squares S(4,4)	82.108	0.686	0.345	0.687
Correlation S(5,-5)	39.239	25.122	0.023	0.035
**RUN-LENGTH MATRIX PARAMETERS**				
Short run emphasis, 90°	10.659	12.516	0.001	<0,001
Grey level nonuniformity, 45°	15.649	11.529	0.001	<0,001
**ABSOLUTE GRADIENT PARAMETERS**				
Mean	18.036	44.271	0.002	0.001
Skewness	63.599	15.598	0.046	0.007

Texture parameters are given in rows. In the columns R&R repeatability and reproducibility of total, and Wilcoxon test for fat saturation series grouped with image slice thickness less than 8 mm, and 8 mm or thicker.

R&R inverted ratio and the small difference between values are associated with poor results in Wilcoxon test with certain exceptions. Comparisons between first and third imaging points achieved significant Wilcoxon test p-values most consistently: within T2-weighted images in both slice thickness groups, and within T1-weighted images in the group of thinner slices. Features ranked in T1-weighted image data were tested in T2-weighted image data and vice versa. These tests with ranked features transposed with T1- and T2-weighted image groups lead to statistically relevant p-values in thinner T1-weighted images and all images in T2-weighted group. In the analyses of first and second imaging timepoints thin slices in general achieved poorer separation than thick slices. Between the second and third imaging sessions Wilcoxon test gave an unsatisfactory result in T1-weighted group. This trend can be seen in the B11 classification results in the framework of T1-weighted images, while the T2-weighted image analyses in B11 show better classification between second and third than first and second imaging points. The best overall discrimination between imaging timepoints in T1-weighted images was given by the run-length matrix parameters describing grey level non-uniformity, run-length non-uniformity, short-run emphasis and fraction of image in runs in one or more directions calculated (horizontal, vertical, 45 degrees and 135 degrees). In the framework of T2-weighted image analyses best the performers were absolute gradient mean and grey level non-uniformity There were some scattering in well acquitted parameters between sub analyses.

Mann-Whitney test was performed for all texture features ranked 1–5 in any classification sub-analysis separately in T1- and T2-weighted images and further subgroups according to slice thickness to analyze differences between stage of malignity (low vs. high/intermediate) and between subjective change of symptoms (unchanged vs. relieved). These analyses did not yield any relevant and consequential additional information on the relation of texture features to grouping parameters.

## Discussion

The goals of this study were show that a) MRI texture analysis can be used in NHL chemotherapy response evaluation b) statistical tests Wilcoxon paired test and R&R can be used to evaluate the separability of texture parameters used to describe textural changes in NHL.

Limitations of our study may be the non-standardized MRI sequence protocols within intra and inter patient images and the use of different slice thickness due to imaging in clinical practice, where patient's clinical stage and the size of the tumor were taken into account when setting imaging parameters. However, multicenter studies on MRI TA have shown transferability of TA parameters achieved from MRI images obtained at different MRI centers with own acquisition parameters [16,38].

To achieve new clinical relevant information by means of texture analysis, the texture changes should come out at the same or earlier timepoint as other quantitative measures of tumor response, for example decrease in tumor volume. The RECIST and WHO criteria for evaluating tumor response in one- or two-dimensional (diameter and product) tumor size is equivalent to a 65% decrease in tumor volume [1]. In this study we calculated tumor size decrease in a short time period: before and after the first cycle of chemotherapy. There are no commonly used criteria for early response assessment using volumetric analysis for use as early in the therapy course as our volumetric evaluation was performed. Considering this, we can use the volumetric results as indicative of early imaging based evaluation of response, not to meet response, and also accept tumor volume decrease percentages smaller than 65% as consequential decrease in tumor size. However, in lymphomas, final clinical response evaluation should include other clinical tests according to [5,6].

Wilcoxon test showed encouraging values in the analyses of E1 and E3, including transferability of feature sets between T1- and T2-weighted images. This confirms our recent results with smaller patient data MaZda texture analysis of combination of T1- and T2-weighted images in single analysis [32].

Our study show that the statistical and autoregressive model texture parameters of MRI data can be successfully tested one by one with Wilcoxon paired test and Gage Repeatability and Reproducibility test to assess the impact of parameter separability in evaluating chemotherapy response in lymphoma tissue. Our results strengthen the applicability of Fisher and POE+ACC methods used in MaZda for automatic feature selection, and also confirm the suitability of the raw parameters in statistical tests. This indicates that raw parameters may be used in analyses other than LDA, NDA and PCA tests to acquire classification.

We have shown that texture parameters change during tumor response to chemotherapy. Comparing initial imaging to the second imaging timepoint, just after the first chemotherapy cycle, there were not such clear changes as at the third imaging timepoint, after four cycles of chemotherapy. The difference in texture appearance between staging and the third imaging timepoint was distinct and emerged from the results of other combinations in both T1-weighted and T2-weighted image types. There might have been better separation in texture features between diagnostic and first evaluation stage if standardized imaging sequence had been used. Our non-standardized MRI sequence may lead too heterogeneous TA features to exactly describe subtle changes in lymphoma tissue in extremely early stages of therapy response evaluation. We still cannot state the importance of subtle textural changes in early response assessment in comparison to volumetric changes in the same time intervals. Further, as controls for examined NHL masses no normal lymph nodes neither NHL masses after treatment were analyzed, since their small size leading to not exact differentiation from surrounding soft tissue structures in MR images.

The response evaluation of lymphomas under treatment using radiological imaging methods is connected strongly with tumor dimensions, instead when using positron emission tomography, tumor lesion activity of tracer uptake is measured. Both methods have certain advantages and disadvantages; major disadvantages related to sensitivity to differentiate residual masses and inflammatory processes from active disease. Functional responses for nocicepti stimuli and antivascular therapy have been detected in recent MRI TA studies [18,31]. In this context changes in textural appearance in MRI during the treatment process probably reflect chemotherapy induced changes in cellular proliferation.

In treatment with a curative orientation it is essential to get early an estimate of response to determine further treatment. MRI texture analysis may provide new insight to be used alone or in combination with other tools in diagnostics and response monitoring of non-Hodgkin lymphomas.

## Conclusion

In conclusion NHL tissue MRI texture imaged before treatment and during chemotherapy can be correctly classified. Our results show promise for texture analysis as a possible new quantitative means for evaluating NHL response. Statistical and autoregressive model texture parameters of MRI data can be successfully tested with Wilcoxon paired test and Gage Repeatability and Reproducibility test to assess the impact of the parameters separability in evaluating chemotherapy response in lymphoma tissue.

## Competing interests

The authors declare that they have no competing interests.

## Authors' contributions

HP, RJ, PLIKL, HJE and PD designed and coordinated the TRE-project. LCVH designed this study, PLIKL, HJE, PD and SS participated in its coordination. LCVH performed the texture data collection and classification, and drafted the manuscript. TL performed statistical analyses. TOS performed the volumetric analysis. TTH designed and made the application for volumetric analysis. All authors participated in manuscript modification, read and approved the final manuscript.
